# Complete genome sequence of *Clostridium botulinum* TMIPH-CB191 (GTC 18335) isolated from a human in Japan

**DOI:** 10.1128/mra.00587-26

**Published:** 2026-07-02

**Authors:** Masahiro Hayashi, Yoshinori Muto, Chie Monma, Jun Yonetamari, Kaori Tanaka

**Affiliations:** 1 Institute for Glyco-core Research iGCORE, Gifu University, Gifu City, Gifu, Japan; 2 Division of Anaerobe Research, Life Science Research Center, Gifu University, Gifu City, Gifu, Japan; 3 Gifu University Center for Conservation of Microbial Genetic Resource, Gifu, Japan; 4 Jumonji University 34760, Niiza City, Saitama, Japan; 5 Division of Clinical Laboratory, Gifu University Hospital 476117, Gifu, Japan; 6 United Graduate School of Drug Discovery and Medical Information Sciences, Gifu University, Gifu City, Gifu, Japan; Fluxus Inc., Sunnyvale, California, USA

**Keywords:** *Clostridium botulinum*, genomes, anaerobes

## Abstract

In this announcement, we report the complete genome sequence of *Clostridium botulinum* TMIPH-CB191 (GTC 18335) isolated from a human in Japan, comprising a circular chromosome and a circular plasmid of 3,954,667 and 5,926 bp, respectively.

## ANNOUNCEMENT

*Clostridium botulinum* is a gram-positive, spore-forming, and anaerobic bacterium of the family *Clostridiaceae* (1, 2). Complete genome sequences of Japanese *Clostridium botulinum* type A isolates remain limited. Therefore, we determined the complete genome sequence of a historical clinical type A strain isolated from a human in Tokyo, Japan (35.6762° N, 139.6503° E), in 1996. The isolate was preserved in a vial containing 20% glycerol and stored at −80°C until further analyses. The strain TMIPH-CB191 was maintained on AccuDia GAM Agar (Shimadzu Diagnostics Corporation, Japan) at 37°C for 48 h under anaerobic conditions (82% N_2_, 10% CO_2_, and 8% H_2_). Colonies were scraped, suspended in Tris-EDTA buffer, and pelleted via centrifugation (10,000 × *g* for 5 min). Genomic DNA was extracted using the NucleoBond HMW DNA kit (MACHEREY-NAGEL, Japan) following the manufacturer’s instructions. DNA concentration and purity were assessed using a NanoDrop spectrophotometer.

The genome was sequenced using PromethION 24 (Oxford Nanopore Technologies [ONT], Oxford, UK) for long-read sequencing and NextSeq 1000 (Illumina, San Diego, CA, USA) for short-read sequencing (3–5). The same DNA extract was used for both platforms. A library was constructed with a Native Barcoding Kit (SQK-LSK114-24, ONT) without shearing for long-read sequencing. Small DNA fragments were removed using the Short Read Eliminator XS Kit (Pacific Biosciences of California, Inc., USA). Sequencing was performed on a PromethION 24 (ONT) with a FLO-PRO114M flow cell. Base calling was performed with Dorado (v7.11.2) (6) (super-accurate base calling), and raw reads were trimmed and quality-filtered using NanoFilt (v2.7.1) (7) with parameters “-l 1000 -q 10 --headcrop 50.” For short reads, the DNA Prep (M) Tagmentation Kit (Illumina) was used for library construction. The quality and quantity of the prepared library were assessed using a Qubit Fluorometer (Thermo Fisher Scientific, Japan) and a capillary electrophoresis system. Subsequently, 2 × 151 bp paired-end sequencing was performed using NextSeq 1000. Reads were processed with fastp (v0.20.1) (8) using “-q 30 -n 20 -t 1 -T 1.” Short- and long-read quality checks were conducted using fastp (8) and NanoPlot (v1.32.1) (7), respectively. Short reads (over 93.8% of bases >Q30) and long reads (mean read quality of 22.0) were assembled using Unicycler (v0.4.8) (9) with default settings. Assembly circularization was performed automatically in a Unicycler. The contig graph was visualized with Bandage (v0.8.1) (10), and the taxonomic integrity was confirmed using BlobTools (v1.0) with short reads (11). The genome was annotated using DDBJ Fast Annotation and Submission Tool (DFAST) (12). The assembly was rotated to start with *dnaA* on the forward strand. Plasmid sequences were analyzed using MOB-typer and MOB-recon tools in MOB-suite (v3.1.9) (13).

Table 1 summarizes the genomic information. Unicycler and Bandage indicated a complete circular genome. Quality and genome statistics were computed using CheckM (v1.2.2) (14) and SeqKit (v2.9.0) (15). CheckM confirmed that the genome was 99.49% complete, with 0.17% contamination. DFAST (12) predicted 3,670 coding sequences, 27 rRNAs, 80 tRNAs, and 3 CRISPR sequences. The botulinum neurotoxin (BoNT) gene was located on the chromosome. The plasmid contained a MOBV relaxase and was predicted to be mobilizable (13).

**TABLE 1:** Information on the complete genome sequence of a *Clostridium botulinum* TMIPH-CB191 strain isolated from a human in Japan

Parameters	Strain name ***Clostridium botulinum*** TMIPH-CB191
NextSeq sequencing^*a*^	
No. of reads	5,280,870
Size (kb)	790,403.1
Average coverage (×)	199.9
DRA accession no.	DRR918816
ONT sequencing^*a*^	
No. of reads	26,474
Size (kb)	500,003,786
Average read length (bp)	18,887
Average coverage (×)	126.4
N50	8,497
DRA accession no.	DRR918815
Assembly	
Assembly N50 (bp)^*c*^	3,954,667
Estimated genome completeness (%)^*d*^	99.49
Estimated genome contamination (%)^*d*^	0.17
Genome structure	One chromosome and one plasmid
DDBJ/GenBank accession nos.	AP046553 (TMIPH-CB191) AP046554 (pTMIPH-CB191)
Genome size (bp)	3,954,667 (TMIPH-CB191) 5,926 (pTMIPH-CB191)
GC content (%)	
Chromosome/plasmid name	28.2 (TMIPH-CB191) 25.6 (pTMIPH-CB191)
No. of coding sequences^*b*^	3,670
No. of rRNAs^*b*^	27
No. of tRNAs^*b*^	80
No. of CRISPRs^*b*^	3

^*a*^ DRA, DDBJ Sequence Read Archive.

^*b*^ DFAST, DDBJ Fast Annotation and Submission Tool.

^*c*^ Determined with SeqKit v2.9.0.

^*d*^ Determined with CheckM v1.2.2.

## Data Availability

The genome sequence of *Clostridium botulinum* TMIPH-CB191 has been deposited into the DNA Data Bank of Japan (DDBJ) (https://www.ddbj.nig.ac.jp/index.html) under accession numbers AP046553 and AP046554. Raw sequence data for TMIPH-CB191 have been deposited into the DDBJ/Sequence Read Archive under accession numbers DRR918815 and DRR918816.
